# DNA-Based Gold Nanoparticle Assemblies: From Structure Constructions to Sensing Applications

**DOI:** 10.3390/s23229229

**Published:** 2023-11-16

**Authors:** Mo Xie, Jinke Jiang, Jie Chao

**Affiliations:** State Key Laboratory of Organic Electronics and Information Displays & Jiangsu Key Laboratory for Biosensors, Institute of Advanced Materials (IAM), Jiangsu National Synergetic Innovation Center for Advanced Materials (SICAM), Nanjing University of Posts & Telecommunications, 9 Wenyuan Road, Nanjing 210023, China; iammxie@njupt.edu.cn (M.X.); iamjkjiang@163.com (J.J.)

**Keywords:** DNA strands, DNA nanostructures, gold nanoparticle, plasmonic, sensing

## Abstract

Gold nanoparticles (Au NPs) have become one of the building blocks for superior assembly and device fabrication due to the intrinsic, tunable physical properties of nanoparticles. With the development of DNA nanotechnology, gold nanoparticles are organized in a highly precise and controllable way under the mediation of DNA, achieving programmability and specificity unmatched by other ligands. The successful construction of abundant gold nanoparticle assembly structures has also given rise to the fabrication of a wide range of sensors, which has greatly contributed to the development of the sensing field. In this review, we focus on the progress in the DNA-mediated assembly of Au NPs and their application in sensing in the past five years. Firstly, we highlight the strategies used for the orderly organization of Au NPs with DNA. Then, we describe the DNA-based assembly of Au NPs for sensing applications and representative research therein. Finally, we summarize the advantages of DNA nanotechnology in assembling complex Au NPs and outline the challenges and limitations in constructing complex gold nanoparticle assembly structures with tailored functionalities.

## 1. Introduction

Precious metal nanoparticles have a wide range of applications in the fields of optics, electronics, catalysis, biomedicine and sensing due to their large specific surface area and abundance of free electrons [1,2,3]. Among them, gold nanoparticles (Au NPs) have been favored by most researchers because of their controllable physicochemical properties. For instance, Au NPs are suitable for surface functionalization, and they can be coupled with a variety of functional materials, including ligands, DNA, proteins, polymers, etc. [4,5,6,7]. In addition, Au NPs have a unique local surface plasmon resonance (LSPR) property [8] and show excellent performance in labeling, imaging and sensing, etc. This property depends on the particle size, shape and aggregation state. Moreover, Au NPs have satisfactory chemical stability and excellent biocompatibility [9]. Au NPs can be surface-modified to increase the interactions with biomolecules, without significant toxic effects on organisms, thus bringing a high degree of effectiveness to the biomedical field. More importantly, the assembly of homogeneous or heterogeneous gold nanoparticles will bring about properties that differ from those of monodisperse nanoparticles. The variations in interactions between nanoparticles due to differences in the particle type, number, relative position, etc., make them an excellent platform for use in designing sensors [10,11,12]. However, the question of how to precisely manipulate the assembly of Au NPs to construct aggregates with specific structural characteristics and predictable properties has become a major research topic.

To date, various strategies have been used to organize monodisperse Au NPs, such as changing the solution environment, adopting amphiphilic block copolymer packaging, electrostatic interactions, etc. [13,14,15,16]. However, these strategies may lead to the over-assembly or agglomeration of Au NPs due to the lack of regulation of the amount, location and specificity of the functionalization of the Au NP surface. Consequently, it is crucial to improve the ability to manipulate individual nanoparticles. Owing to their programmability, DNA molecules can be encoded into highly specific sequences, providing a solid foundation for predictable assembly. Thus far, DNA has been successfully used to construct DNA nanostructures of various shapes, sizes and dimensions [17,18,19]. In addition, DNA molecules can be attached to the surfaces of Au NPs through Au-S bonds or poly-adenine (poly-A) chemical attachment, which have been proven to be an ideal material for the controllable assembly of nanoparticles [20,21]. In DNA nanostructures, the positional accuracy of DNA sequences can be used to position and assemble various functional materials (such as small molecules, proteins, polymers and metal nanoparticles) with nanoscale accuracy [22].

In this paper, we review the progress of DNA-based superior assemblies of Au NPs and their applications in the field of sensing in the past five years. Firstly, we highlight the role of DNA strands and the DNA structure in guiding the assembly of Au NPs and the latest progress. Then, we describe the application of gold nanoparticle aggregates based on DNA assembly in the sensing field (including fluorescence-based sensing, chiral sensing, SERS-based sensing, etc.) and the representative research. Finally, we discuss the advantages of DNA nanotechnology in improving the assembly complexity of Au NPs, and we outline the challenges and limitations of building complex gold nanoparticle assembly structures with custom functions.

## 2. DNA-Based Assembly of Gold Nanoparticles

### 2.1. Gold Nanoparticle Multimers

The properties of gold nanostructures are related to their shape, size and spatial arrangement. Assembling gold nanoparticles with well-defined structures and quantities in a specific space enables the specific amplified modulation of electronic, magnetic and optical signals. Owing to the unique self-assembly ability of DNA, gold nanoparticles can be organized in a highly precise and controllable way by precisely designing DNA sequences or DNA nanostructures to obtain Au NP multimers with well-defined structures and functions. In this section, we will focus on DNA strands and DNA structures mediating the construction of gold nanoparticle assemblies.

#### 2.1.1. DNA Strand-Mediated Assembly

**Figure 1:**
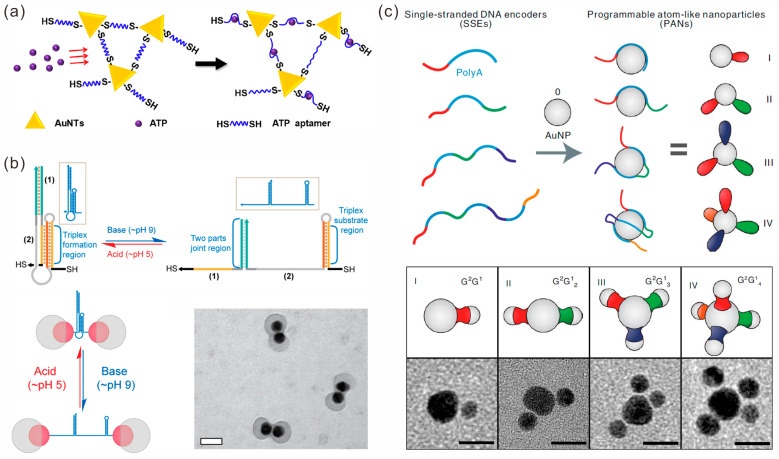
DNA strand-mediated assembly of gold nanoparticles. (**a**) Gold nanotriangles assemble gold porous structures under the action of dithiol adenosine triphosphate aptamer for ATP detection. Reprinted with permission from ref. [24]. (**b**) pH-responsive dimer assembly by using DNA-modified selectively encapsulated gold nanoparticles. Scale bar, 50 nm. Reprinted with permission from ref. [28]. (**c**) Patterning gold nanoparticles with single-stranded DNA encoders containing poly-A, used for the production of programmable atomic equivalents. Scale bar, 10 nm. Reprinted with permission from ref. [31].

Coupling thiol-modified DNA molecules onto the surfaces of Au NPs through Au-S bonds is a common approach to functionalizing Au NPs. Liang et al. designed and modified complementary single-strand DNA sequences on AuNPs with diameters of 200 nm and 40 nm, respectively. Then, core-satellite nanostructures were formed through base complementary pairing hybridization. Subsequently, further silicification yielded a stable nanocavity called the DNA-silicified template for a Raman optical beacon (DNA-STROBE) model [23]. This structure provides a universal method for ultrasensitive label-free sensing exploration. In addition to monothiol DNA modification and hybridization self-assembly strategies, the dithiol DNA strand cross-linking strategy can also achieve the organization of Au NPs. Liu et al. employed a dithiol adenosine triphosphate (ATP) aptamer as a cross-linking molecule and assembled a porous cross-linking structure of gold nanotriangles (AuNTs) at the tip of the capillary to construct a SERS-electrochemistry nanopore platform for ATP detection, which showed higher SERS activity than that of conventional gold nanoparticles (Figure 1a) [24].

However, in the above strategies, thiol-modified DNA sequences are nonselectively chemically attached to the surfaces of Au NPs at high densities, which may lead to the uncontrollable positioning and number of Au NPs in further assembly. Researchers have proposed multiple strategies to control the placement of DNA sequences at specific positions on Au NPs, thereby achieving the controllable assembly of Au NPs. Coughlin et al. proposed a light-mediated method to selectively release single-stranded DNA on the resonance region of the localized surface plasmon (LSP) from the surfaces of gold nanostars (AuNS) [25], followed by the functionalization of another thiol-DNA to this region, thereby achieving the functionalization of different DNA sequences at the main body and tip of the AuNS structure for the assembly of more complex structures. He et al. proposed a cold-driven approach that utilizes the repulsion of locally high concentrations of DNA by the crystallization of water molecules to precisely control the DNA coupling density on the surfaces of spherical gold nanoparticles (AuNPs) to obtain dimers, trimers and core-satellite structures [26]. The method is simple and requires only one step, without reagents such as salts, acids and surfactants, which can avoid complicated post-processing. In addition, anisotropic Au NPs with different coverage rates can be prepared by the eccentric encapsulation of Au NPs, and functionalization can be carried out in a site-specific way, achieving the specific and controllable modification of DNA [27]. Gibson et al. adopted this strategy to obtain anisotropic gold nanospheres and nanorods [28]. Then, they connected the two nanoparticles into dimers by the complementary hybridization of DNA strands with three-helical partial fragments. By adjusting the pH, the distance between nanoparticles can be changed, thereby achieving the regulation of the plasmonic coupling between dimers (Figure 1b). In addition to regulating the surface coverage of Au NPs, bridging-mediated strategies have been used to guide the assembly of Au NPs. Zhang et al. designed a bridge DNA structure consisting of one double-stranded midsection and four single-stranded tails, and AuNPs could be connected with the single-stranded tails [29]. They achieved the dynamic regulation of one-step and multi-step reactions through toehold-mediated strand displacement reactions.

Due to the high cost of thiol-modified DNA, the use of poly-A DNA for functionalized Au NPs has received increasing attention. Ye et al. investigated the interaction between poly-A DNA and AuNP [30]. It was shown that, firstly, the loading density of DNA on the AuNP surface was almost independent of the length of poly-A. Secondly, compared with salt aging and low pH methods, freezing has higher biological interface stability. In addition, the formation of poly-A duplexes after freezing results in enhanced fluorescence. Based on these studies, the author utilized the freeze-driven assembly of plasmonic dimers using DNA with poly-A sequences at both ends. Fan and colleagues conducted an in-depth study on the role of ploy-A DNA in mediating the formation of AuNP assemblies. In 2020, they proposed a strategy for the patterning of gold nanoparticles [31]. They designed single-stranded encoders (SSEs) using specific single-stranded DNA containing poly-A sequences and synthesized a series of programmable atom-like nanoparticles (PANs) with precisely controllable valence states (Figure 1c). Further, they designed specific assembly and hybridization reactions to prepare “colloidal molecules” with different sizes, compositions and chirality, and they realized bond breaking, bonding, rearrangement and logical operations through reversible reconstruction. The following year, they utilized the affinity between poly-A and the surfaces of AuNPs to extend anti-miRNA domains onto the surfaces of AuNPs to construct spherical nuclear acids (SNAs) [32]. By adjusting the length of poly-A to control the number and conformation of anti-miRNAs on AuNPs, and further assembling AuNP-anchored miRNAs, they evaluated the capture efficiency of SNAs with target miRNAs.

#### 2.1.2. DNA Tile-Mediated Assembly

**Figure 2:**
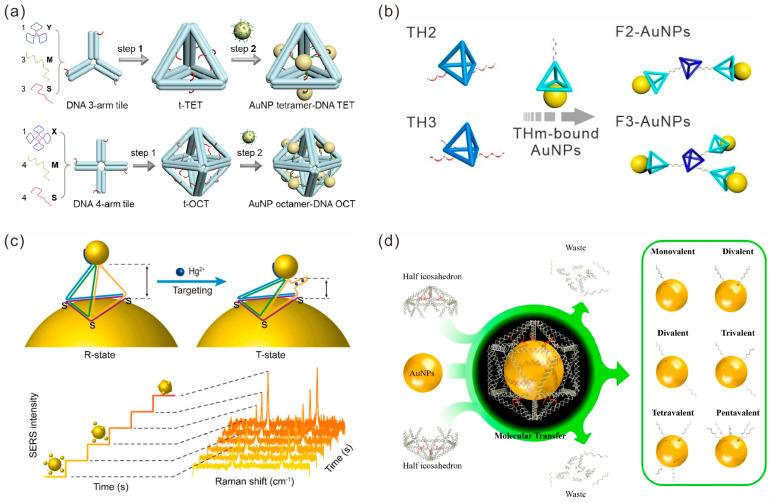
DNA tile-mediated assembly of gold nanoparticles. (**a**) DNA tile-assembled polyhedral nanocages were used to construct well-defined AuNP cluster–DNA cage hybrids. Reprinted with permission from ref. [34]. (**b**) DNA tetrahedral conjugated AuNPs as building blocks to form specific AuNP clusters. Reprinted with permission from ref. [35]. (**c**) DNA tetrahedron-based core-satellite structures as SERS molecular rulers to detect conformational changes triggered by Hg^2+^. Reprinted with permission from ref. [37]. (**d**) Three-dimensional molecular transfer strategy for preparation of DNA-printed nanoparticles (DPNPs) with controllable number and spatial position of DNA strands. Reprinted with permission from ref. [41].

Self-assembly based on the DNA tile structure is one of the most important parts of structural DNA nanotechnology. Although designing DNA tiles typically only requires a few simple DNA strands, it is possible to assemble precise DNA nanostructures ranging from simple to complex. Additionally, the combination of functional materials with DNA nanostructures to form complexes with well-defined compositions and configurations will lead to new properties and applications [33]. As an example, Yu et al. reported a simple AuNP cluster-DNA nanocage hybrid assembly method [34]. They first constructed DNA tetrahedral and octahedral structures using DNA three-arm tiles or DNA four-arm tiles, respectively. Subsequently, the overhangs on a polyhedron hybridized with the complementary DNA-modified AuNPs allowed AuNPs to be anchored at specific locations to directly assemble AuNP tetramer–DNA tetrahedron hybrids and AuNP octamer–DNA octahedron structures (Figure 2a).

In addition to individual DNA tile nanostructures, high-order assemblies of multiple DNA tile structures have also been used to guide the formation of Au NP multimers. He et al. used a DNA tetrahedron with a thiol-anchor constructed by four DNA single strands stoichiometrically conjugated with AuNPs, resulting in monovalent AuNPs [35]. Furthermore, monovalent AuNPs were used as building blocks to construct a specified number of high-order AuNP clusters through assembly (Figure 2b). Tan et al. utilized the DNA tetrahedron structure to control the localization and alignment of AuNPs of three different sizes (5 nm, 10 nm, and 20 nm in diameter, respectively) to assemble a strongly coupled plasmonic core-satellite structure, which was called the TDN-based AuNP core-satellite plasmonic nanostructure (TetrAuCS) [36]. Feng et al. designed a DNA tetrahedron in which one edge was a single-stranded DNA aptamer that could recognize and respond to Hg^2+^. Three vertices of the DNA tetrahedron were anchored to a core AuNP with a diameter of 100 nm through Au-S bonds, and another vertex was connected to a satellite AuNP with a diameter of 20 nm through poly-A to construct a core-satellite nanostructure (Figure 2c). This structure served as a SERS molecular-ruler to detect conformational changes under Hg^2+^ stimulation [37].

The functionalization of individual DNA strands at specific sites on the surfaces of nanoparticles can greatly improve the structural diversity. Pattern transfer strategies [38,39,40] can inherit the DNA molecular information encoded in the template with high fidelity, achieving control over the number, direction and length of functionalization on particles. Xie et al. demonstrated a three-molecular transfer strategy [41]. They first encapsulated a AuNP in a DNA icosahedron cage (I-Cage) template; then, the DNA strands predesigned in the nanocages could be covalently coupled to the AuNP surface through Au-S bonds. Subsequently, by removing the I-Cage template, the predesigned DNA patterns were transferred to the surfaces of AuNPs to obtain atomically equivalent DNA-printed nanoparticles (DPNPs) and further used for the assembly of satellite nanostructures (Figure 2d).

#### 2.1.3. DNA Origami-Mediated Assembly

DNA origami [42] is a technique that uses a long ssDNA called a scaffold and a set of short ssDNA called staples to construct DNA nanostructures. Each staple is unique and can be assembled to a specific position on the scaffold, which results in the addressability of DNA origami. Functional materials such as metal nanoparticles, quantum dots, fluorescent molecules and proteins can be positioned with nanoscale accuracy on DNA origami [43,44,45,46]. Furthermore, the arrangement of these functional materials on DNA origami is not limited to static systems, and the dynamic manipulation of functional materials in spatial and temporal can also be easily achieved [47], which expands the range of DNA-driven gold nanoparticle assemblies.

**Figure 3:**
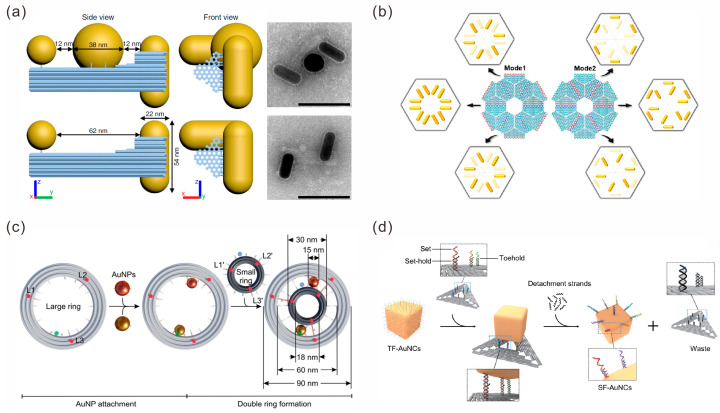
DNA origami-mediated assembly of gold nanoparticles. (**a**) DNA origami-based assembly of NR-NR and NR-NS-NR chiral structures. Scale bar, 100 nm. Reprinted with permission from ref. [49]. (**b**) DNA origami hexamer templates used to construct three types of bi-star and three types of pinwheel AuNR chiral supramolecular structures. Reprinted with permission from ref. [50]. (**c**) The construction of a rotary nanodevice. Reprinted with permission from ref. [55]. (**d**) DNA origami used as templates to transfer patterns onto AuNCs. Reprinted with permission from ref. [56].

DNA origami has been widely used in static assembly systems for Au NPs. Yeşilyurt et al. constructed a meta-emitter that was composed of three AuNPs and a fluorophore [48]. They extended two groups of capture strands on one side of the triangular DNA origami, while they extended a third group of capture strands on the other side to assemble three AuNP assembly nanoantenna and obtained a gap of about 3 nm. Fluorophore ATTO 647N was positioned at the hotspot of the gap, and a plasmonic trimer nanoantenna driven by a single dye molecule was constructed to realize unidirectional meta-emitters. Martens et al. connected gold nanorods (AuNRs) on the two ends of a 3D DNA origami with a distance of 62 nm, forming a 90° heteromorphic nanorod–nanorod (NR-NR) chiral sample. A 40 nm gold nanosphere (AuNS) was attached in the middle of two AuNRs to form a nanorod–nanosphere–nanorod (NR-NS-NR) chiral structure (Figure 3a) [49]. These chiral arrangement structures could be treated as a coupled electron oscillator system that exhibited complex chiral optical fields, resulting in a strongly enhanced CD response.

In order to construct more complex assemblies of Au NPs, DNA origami is also used as assembly blocks to construct static high-order gold nanostructures. Liu et al. proposed a strategy for the 2D thin-layered chiral supramolecular self-assembly of programmable AuNRs guided by DNA origami hexamers [50]. They designed capture strands at defined positions on the triangular DNA origami and used specific connector strands to splice the DNA origami into different hexamer templates. AuNRs as building blocks were captured by the template-specified positions through the modified complementary DNA strands, constructing three types of bi-star and three types of pinwheel AuNR chiral supramolecular structures (Figure 3b). The structures exhibited strong anisotropy and chiroptical responses. The following year, they adopted the same strategy to further create chiral core-satellite nanoparticle superstructures on triangular DNA origami hexamer templates by using one spherical gold nanoparticle as the core and six gold nanorods as satellites. A split or nonsplit circular dichroism (CD) line shape optical activity state was produced by the different conformation structures under light spin excitation [51]. In addition to the above strategy of specifically capturing gold nanoparticles on the templates of DNA origami higher-order structures, it is also a common method to assemble an individual DNA origami–gold nanoparticle composite structure as a building block before further assembling the high-order structure. For instance, Zhou et al. first assembled an AuNP in the cavity of a 3D hexagonal prism DNA origami (HDO) and then constructed symmetric or asymmetric AuNP multimers through orthogonal and directional bonding [52].

Introducing dynamic DNA assembly into structural systems can confer tailored optical properties to gold nanoparticle assemblies. Liu‘s group conducted an in-depth study. In 2019, they assembled multilayer sliding nanosystems by combining three DNA origami filaments with two AuNPs at different levels [53]. The sliding nanosystem could perform coordinated and reversible sliding motions driven by DNA fuel strands. Simultaneously, AuNPs can be used as optical probes to dynamically interact with fluorophores anchored on DNA origami to detect the sliding process in situ. In 2021, they designed a double-layer plate DNA origami template with a curved track fixed on the top of the plate to anchor a AuNR to the rotary module, and a AuNR walking module was assembled on the bottom surface of the plate [54]. Rotating and sliding motions were performed by toehold-mediated strand displacement reactions. In 2022, they designed a large DNA origami ring with an inner diameter of about 60 nm as the ring gear and a small DNA origami ring with an outer diameter of 30 nm as the sun gear; they then assembled two AuNPs with a diameter of 15 nm in the gap as the planet gears to form a rotary nanodevice (Figure 3c) [55]. Fluorescence spectra were used to record the optical rotation dynamics of the nanodevice in real time.

Similar to DNA tiles, DNA origami can also be used as a template to transfer patterns onto AuNPs. Niu et al. reported a DNA origami-based nanoprinting (DOBNP) strategy to transfer basic DNA strands of pre-determined sequence and location from triangular DNA origami to the surfaces of gold nanocubes (AuNCs) [56]. These DNA strands ensured a specificity connection between the AuNCs and AuNPs, resulting in the generation of a stereo-controlled AuNC–AuNP nanostructure (AANs) with a controlled composition and geometry (Figure 3d). They anchored individual dye molecules in hotspot regions and detected the stronger amplification of surface-enhanced Raman scattering (SERS) signals due to significantly enhanced electromagnetic fields. The method provides an opportunity to fabricate stereo-controlled metallic nanostructures for the design of highly sensitive photonic devices. Additionally, Xiong et al. developed a method called molecular stamping (MOST) to pattern DNA-coated nanoparticles [57]. In this patterning process, the coordinated DNA frame acts as a molecular stamping apparatus (MOST App) that transfers and immobilizes a DNA sequence (named molecular “ink”) to the surface of the nanoparticle to form a presetted pattern. After the nanoparticles are treated by the MOST process, the surfaces of the nanoparticles have single-molecule “patches” as anisotropic “bonds” with different affinities. Further, these nanoparticles are assembled into predefined clusters whose structure is determined by the location of the patches.

### 2.2. Gold Nanoparticle Arrays

When arranged into arrays, the strong interactions between periodic nanoparticles provide a unique opportunity to realize materials with interesting and unusual photonic and metamaterial properties [58]. In this section, we will discuss the assembly of 1D, 2D and 3D nanogold arrays under the guidance of DNA strands and DNA nanostructures.

#### 2.2.1. 1D Arrays

**Figure 4:**
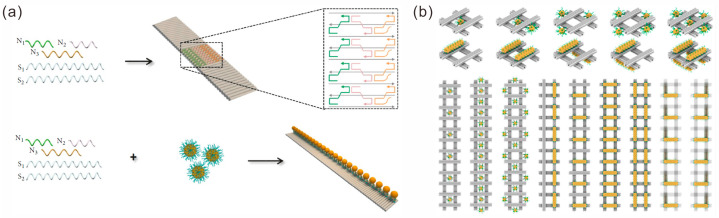
Construction of 1D arrays of gold nanoparticles. (**a**) Assembly of 1D ribbon-like DNA nanostructure and formation of 1D AuNP lines. Reprinted with permission from ref. [60]. (**b**) Au NP polymers on DNA origami hashtag tile polymer templates. Reprinted with permission from ref. [63].

A typical procedure for preparing 1D arrays usually involves two processes: the assembly of DNA nanostructure templates and the localization of Au NPs. The essential aspect of the procedure lies in the design of stable DNA nanostructure templates. DNA nanostructures with repetitive units assembled by DNA bricks provide a favorable template foundation for 1D nanogold arrays. For instance, Liang et al. assembled 1D DNA structural templates using repeating units composed of three DNA strands, and they used it to assemble an ordered linear AuNP plasmonic nanostructure at the oil–water interface. This structure had excellent specificity for the detection of miRNA-155 [59]. Ren et al. designed three shorter ssDNA sequences (N1, N2 and N3) to fold with two longer ssDNA sequences (S1 and S2) to form a ribbon-like DNA nanostructure with repetitive rectangular units [60]. Five strands, including a capture sequence, N3, were mixed with complementary sequence-modified AuNPs to form 1D plasmonic gold metamaterials (Figure 4a), which could produce enhanced Raman scattering. Golla et al. reported a helically twisted nanoribbon for the further assembly of AuNPs or AuNRs to fabricate 1D chiral plasmonic nanostructures [61]. Zhang et al. designed a complex DNA structure template [62]. They used double crossover (DX) tiles composed of five DNA strands as assembly units, and they designed sticky ends on both sides and ends to assemble bundles with an adjustable width and composition of 1D DNA bundles and large AuNPs.

Benefiting from the excellent programmability and addressability of DNA origami templates, Au NPs can be easily configured into 1D arrays. Wang et al. constructed a highly rigid 1D chain structure formed by DNA origami hashtag tile polymerization, which served as a frame for Au NPs assembly, and they precisely arranged a variety of microscale chiral and magnetic plasmonic polymers (Figure 4b) [63]. In addition to static assembly, dynamic structures have also been designed and fabricated. Johnson et al. designed and assembled a DNA origami hinge structure with a AuNP inside. When the temperature changed, the structure generated a thermal response, leading to a configuration transition [64]. Then, composite arrays of linear NPs and hinges with variable configurations were obtained by designing connections between the hinge arms and thermal actuator.

#### 2.2.2. 2D Arrays

**Figure 5:**
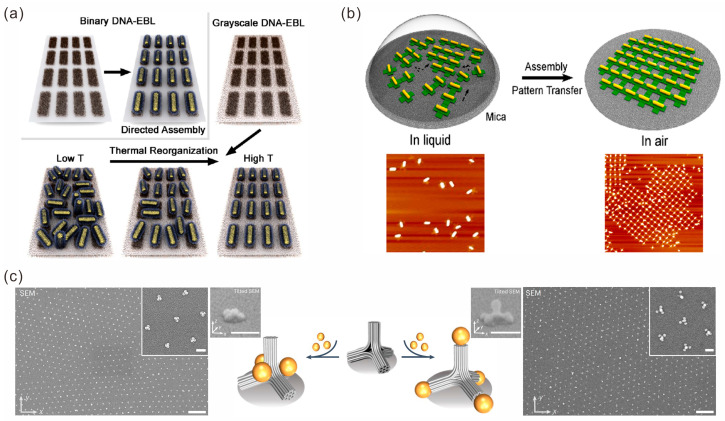
Construction of 2D arrays of gold nanoparticles. (**a**) Temperature-dependent reconfigurable AuNR 2D structure. Reprinted with permission from ref. [67]. (**b**) Cross-shaped DNA origami–AuNR composite as building blocks for assembly of 1D and 2D AuNR arrays. Reprinted with permission from ref. [69]. (**c**) AuNPs assembled on the DNA origami structure and placed on the x-y plane to form a periodic array. Reprinted with permission from ref. [71].

There are two effective traditional methods for the preparation of Au NP 2D arrays: one is to use bonding agents with specific interactions (such as DNA strands) as assembly media, and the other is to use patterns on the substrate as templates to limit the assembly of NPs. Given that DNA-AuNP superlattices extruded between SiNx windows and xTEM liquid cells can produce large amounts of 2D NPs, Shekhirev et al. produced large-area monolayer NP assemblies via the crystallization of a finite volume solution containing complementary DNA-functionalized NPs by weak interactions on a surface [65]. The 2D NP structure could respond to changes in solvent composition. Combining DNA self-assembly with lithography can produce functionalized 2D surfaces for the high-resolution assembly of nanoparticle arrays. Mirkin’s group patterned DNA on a gold-plated substrate using photolithography technology by placing DNA in pores constrained by a polymethyl methacrylate (PMMA) template; they then assembled 80 nm gold cubes with complementary DNA sequences in the holes and finally removed the PMMA template to obtain a 2D array of gold cubes [66]. Using a similar strategy, Myers et al. manufactured structurally reconfigurable metasurfaces of AuNRs [67]. They controlled the position and orientation of AuNRs by controlling the DNA functionalization pattern and drove the reconfiguration of the NP arrays based on the temperature dependence between the NPs and the ssDNA anchoring density (Figure 5a).

However, the methods above require complex surface modification, which hinders their further application. Therefore, it is necessary to develop an effective strategy that does not require special surface treatment. In fact, DNA origami-guided assembly has demonstrated an unparalleled ability to organize metal nanoparticles precisely into their designed locations without special treatment. Shen et al. used DNA origami hexagons as building blocks to design and assemble honeycomb arrays or tubular high-order networks with sizes of up to a few micrometers [68]. Additionally, the addressability of the unit block allowed the researchers to precisely place the Au NPs at the specified positions and obtain DNA origami–nanoparticle composite blocks, which were further assembled to realize the construction of periodic gold nano-arrays. Similarly, Yang et al. assembled 1D and 2D AuNR array structures using cross-shaped DNA origami–AuNR composites as building blocks (Figure 5b) [69].

Bottom-up assembly based on DNA origami can also be combined with top-down lithography [70]. Martynenko et al. demonstrated a method for the precisely targeted placement of various shapes of DNA origami on the micrometer to millimeter scale [71]. They utilized two approaches of connector-mediated binding (hollow tubes) and direct binding (the tetrapod) through self-aligned binding to realize the upright positioning of various DNA origami shapes. As a proof of concept, they assembled the DNA origami structures with AuNPs and ultimately connected individually placed DNA origami with DNA pillars on an x-y plane to create continuous periodic networks (Figure 5c).

#### 2.2.3. 3D Lattices

**Figure 6:**
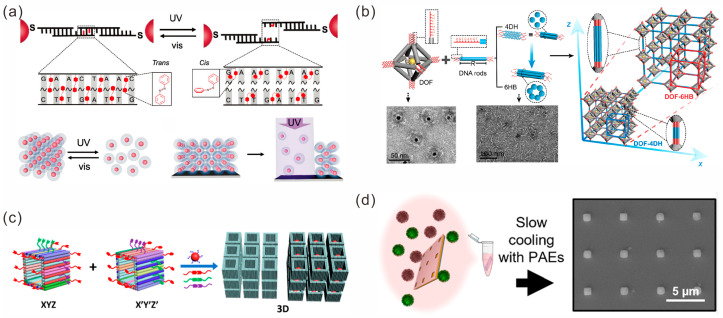
Construction of 3D lattices of gold nanoparticles. (**a**) Photoisomerization of azobenzene molecules during UV and visible light switching can lead to structural transformation of the nanoparticle lattice. Reprinted with permission from ref. [75]. (**b**) DNA rod bridged octahedral DOF/NP complex crystals. Reprinted with permission from ref. [78]. (**c**) 3D semi-closed cuboid DNA nanochambers (DNCs) used to assemble 3D lattices. Reprinted with permission from ref. [86]. (**d**) Preparation of colloidal crystal arrays in microwells. Reprinted with permission from ref. [88].

Programmable DNA-mediated assembly provides a vast and diverse design space for 3D lattice construction, where the nanoparticle size, spacing and crystal symmetry can all be independently controlled. Mirkin’s group developed a method for the construction of lattices with programmable nanomaterials [72,73,74]. Functionalized nanoparticles act as “atoms” and oligonucleotides act as “bonds”, and they are assembled into crystal structures with an adjustable composition, symmetry and lattice parameters, by tightly controlling the assembly characteristics of these oligonucleotide–nanoparticle conjugates. In recent years, they have studied the properties of crystal structures by adjusting the design of DNA bonds based on this construction method. In 2020, they reported a new method for the synthesis of colloidal crystals using azobenzene modified photo-responsive DNA strands [75]. The photoisomerization of azobenzene molecules during UV and visible light switching can lead to the reversible assembly and decomposition of the nanoparticle lattice. By using ultraviolet light as a trigger signal, nanoparticles on colloidal crystal sheets can be selectively removed, enabling them to be photolithographed into specific shapes (Figure 6a). In 2023, they designed a DNA dendrimer as a symmetry-breaking synthon to encode anisotropic and orthogonal interactions on individual colloidal particle building blocks, which broke the symmetry of colloidal crystals [76]. Moreover, they investigated the mechanical strength of metamaterials formed by the DNA-mediated assembly of different nanoparticles. They found that nanosolid, nanocage and nanoframe mechanical metamaterials with the same crystal symmetry exhibited significantly different specific stiffness and strength. In particular, the strength of the nanoframe lattice was about six times stronger than that of the nanosolid lattice [73].

DNA origami guides the controllable arrangement of nanoparticles in a programmable manner, and it has rapidly developed into an ideal method of constructing nanoparticle crystals. Tian’s group has performed a great deal of research in this direction. Firstly, since the vertices and internal cavities of the regular-octahedral DNA origami wireframe structure can be designed with specific connection sequences, AuNPs only need to be modified with complementary sequences, which can be loaded into the octahedral cavity. Drawing inspiration from the “node-and-spacer” construction approach of coordination polymers, they used the octahedral frameworks as “nodes” and ssDNA as “spacers”, and, by regulating the number and spatial positions of ssDNA extended from the vertices of the octahedral shells, the valences and directions of nanoparticles were designed, which was equivalent to achieving the “valence state” encoding of the internal AuNPs [77]. A variety of static and dynamic crystal structures [78,79,80] were obtained by the further assembly of the encoded structures (Figure 6b). Secondly, they introduced an elongated octahedron framework to explore the co-crystallization of anisotropic heterogeneous species and realize the construction of composite superlattices [81,82,83]. Furthermore, DNA structures such as tetrahedrons and cubes were used as frames with valence. AuNPs of different sizes, quantum dots and proteins were loaded inside the frame as nano-objects, and a variety of ordered multivariate heterogeneous superlattices and different crystalline systems were constructed [84,85]. In addition to wireframe DNA origami, Lin et al. used a similar strategy to design and assemble 1D, 2D and 3D arrays by encoding “keys” on the three orthogonal axes of 3D semi-closed cuboid DNA nanochambers (DNCs) (Figure 6c) [86]. Julin et al. employed the high negative charge of the surface of a DNA origami to assemble a six-helix bundle DNA origami and cationic AuNPs into a 3D ordered superlattice [87].

Top-down lithography and bottom-up self-assembly techniques have also been shown to be useful for 3D lattice construction. The preparation of microarrays and the functionalization of the bottoms of pores are performed in the same manner as for the 2D arrays described above. Two groups of nanoparticles with complementary sequences were prepared and hybridized at room temperature. Under the limited template space, the gold nanoclusters were forced to merge and reorganize to produce a highly ordered single crystal superlattice (Figure 6d). The orientation, position and size of the lattice could be controlled by the template holes and the assembly conditions [88].

## 3. Sensing Applications of Gold Nanoparticle Assembly Structures

Owing to the excellent properties of gold nanoparticles, they are ideal for sensing substrates. Sensors constructed with recognition elements (including ligands, fluorescent molecules, quantum dots, nucleic acid aptamers, etc.) have been widely used in optics, imaging, detection and other fields. According to the construction strategies described in Section 2, Au NPs can be precisely designed into sensors with well-defined structural features and predictable performance under the guidance of DNA sequences and DNA structures. In this section, we will introduce some representative applications of DNA-based gold nanoparticle assembly structures in sensing from the perspective of sensor construction strategies and technical methods.

### 3.1. Fluorescence-Based Sensors

The interaction between fluorescent sensors and target analytes leads to changes in the fluorescence signals, so as to realize the qualitative and quantitative identification and analysis of the target analytes. The fluorescent sensor is one of the most commonly used sensing strategies as it is very attractive in terms of high sensitivity and selectivity and real-time detection, and it typically achieves detection limits as low as nanomolar to picomolar, but it is susceptible to the effects of background interference and fluorescence quenching. The interaction between fluorophores and Au NPs is related to the material, shape, size, distance and direction between the dye and NPs [89].

**Figure 7:**
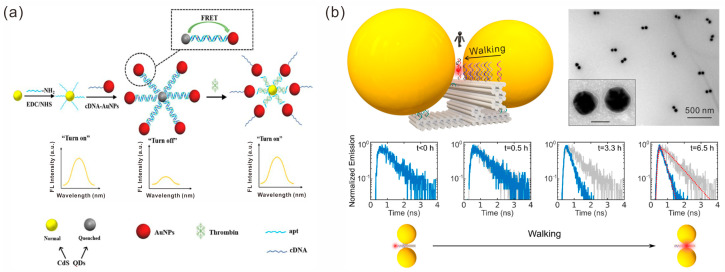
Fluorescence-based sensors. (**a**) The sensing mechanism of an off-on fluorescence aptasensor. Reprinted with permission from ref. [92]. (**b**) Single fluorophore walker devices and in situ monitoring of fluorescence intensity. Reprinted with permission from ref. [99].

When the distance between two fluorophores is close enough, energy exchange between the donor and acceptor fluorophores through dipole interaction occurs, which is called Förster resonance energy transfer (FRET). This property has been widely used in the construction of fluorescence-based sensors [90,91]. For example, Li et al. developed an “off-on” fluorescence aptasensor for thrombin detection based on FRET between donor CdS QDs and acceptor AuNPs [92]. The CdS QDs and AuNPs were tightly coupled due to the hybridization of the modified aptamers and complementary cDNA strands, respectively, resulting in FRET and the fluorescence quenching of donor CdS QDs in the “off” state. After the addition of thrombin and specific binding with the adapter, the AuNPs receptor was isolated, and the fluorescence recovery of the donor CdS QDs displayed the “on” state (Figure 7a).

The interaction between the surfaces of gold nanoparticles and photons produces localized surface plasmon resonance (LSPR) [93], in which the particle–particle coupling generates a “hotspot” region with large LSPR. It has been proven that placing fluorescent molecules in hotspot areas leads to fluorescence enhancement [94,95]. Tinnefeld’s group constructed a variety of nanoantennas by assembling AuNPs on DNA origami via DNA nanotechnology. They studied the interaction between fluorophores and plasmonic hotspots and explored the application of nanoantennas. In recent years, they have investigated the emission directionality of rotating single fluorophores in hotspot regions of statically assembled AuNP dimer structures [96], demonstrating that the emission directionality follows a dipole mode associated with the main resonance mode of the nanoantenna. The advantages of anisotropic AuNRs for fluorescence enhancement were compared with those of isotropic AuNPs [97]. They also explored the application of fluorescence sensors built by nanoantennas to create a platform for specific single-antibody detection through signal amplification generated by DNA origami nanoantennas in combination with unlabeled antibody detection [98]. Different from Tinnefeld’s group, Xin et al. constructed a fluorophore walker device to study the interaction between fluorophores and plasmonic hotspots by driving fluorescence molecules to approach the hotspot along the specified trajectory through DNAzyme–RNA interactions [99]. They found that when the fluorophore approached and finally entered the hotspot of the plasmonic nanoantenna, the fluorescence of the fluorophore decayed at an accelerated rate (Figure 7b). 

### 3.2. Chiral Sensors

Combining chiral recognition and real-time sensing, chiral sensors have the advantages of high sensitivity, real-time performance and high selectivity and specificity. However, they also have the disadvantage of being susceptible to non-specific interactions. Chiral plasma nanostructures exhibit exceptionally strong chiral optical signals, which provide the possibility for asymmetric photophysical and photochemical processes controlled by circularly polarized light [100]. Based on DNA self-assembly technology, the positioning and orientation of Au NPs to create a plasmonic superstructure can achieve polarization-sensitive optical properties and display strong CD signals, thereby constructing chiral sensors. The interaction between the analyte and the sensor triggers the transformation of the chiral structure, leading to changes in the chiral optical signal, achieving highly sensitive detection with a detection limit of nanomolar or lower.

**Figure 8:**
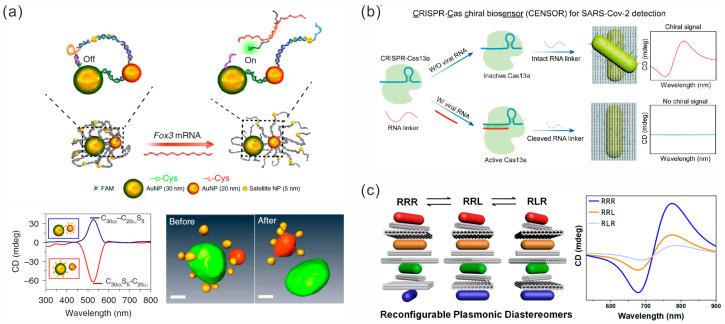
Chiral sensors. (**a**) *Fox3* mRNA triggers the transformation of chiral structure configuration. Reprinted with permission from ref. [102]. (**b**) A chiral biosensor for SARS-CoV-2 detection. Reprinted with permission from ref. [108]. (**c**) Complex reconfigurable plasmonic diastereomers. Reprinted with permission from ref. [110].

The DNA strand-guided assembly of AuNPs of different sizes is one of the strategies used to construct chiral structures [101]. For instance, Qu et al. used ssDNA strands to bridge a 30 nm AuNP and a 20 nm AuNP, and 5 nm NPs were connected to these two AuNPs to form two types of binuclear chiral nanostructures, C_30_S_5_-C_20_ and C_30_-C_20_S_5_, respectively (Figure 8a) [102]. These chiral structures showed an affinity with cytoskeleton protein α-actinin-1, and the optical moment generated by the chiral probe induced by polarized light was as high as 10 nN. The interaction between the mechanical force generated by the optical moment and the cytoskeleton stimulated the differentiation of stem cells, activated the high expression of specific genes of neurons in stem cells and accelerated the differentiation and maturation of neurons. With the differentiation of stem cells, *Fox3* mRNA that complemented the chiral probes was expressed, causing the chiral structure to produce a conformational transformation within the cell, and its CD spectrum signal was reversed. The light response of the chiral probe enhanced the differentiation and maturation of neural stem cells, and this provides a new strategy for the diagnosis and treatment of protein conformational diseases.

Utilizing the addressability of DNA origami to localize the directed assembly of gold nanoparticles is another strategy to accurately modulate chiral structures [103,104]. Among them, anisotropic AuNRs are some of the commonly used assemblies. Govorov and co-workers assembled an AuNR on the different sides of a rectangular origami structure, respectively, and constructed a simple AuNR dimer chiral structure. They used the photothermal [105] and thermionic [106] effects to investigate the chiral photoresponsive system with circular polarization sensitivity. In addition, Ryssy et al. explored the structural reconstruction and pH-dependent CD response of a AuNR dimer chiral structure caused by pH regulation [107]. Yu et al. introduced an RNA linker between a DNA origami and AuNR, which was essential to realize the assembly of chiral biosensors and right-handed chiral signals [108]. The presence of SARS-CoV-2 RNA led to a decrease in the optical chirality signal, thus realizing the quantitative detection of chirality biosensing (Figure 8b). Moreover, complex gold nanoparticle multimeric chiral sensing has been developed [109,110]. Wang et al. proposed a stepwise assembly strategy to construct complex plasmonic diastereoisomers with multiple distinguishable chiral centers, which could generate characteristic CD signals by selectively controlling each chiral center (Figure 8c) [110].

### 3.3. SERS-Based Sensors

Surface-enhanced Raman scattering (SERS) technology is widely used in the field of sensing and detection. The electromagnetic field enhancement effect of precious metal nanostructures significantly amplifies the Raman signals of target molecules adsorbed on SERS substrates [111], making it one of the most commonly used methods of detecting extremely low concentrations (femtomolar to nanomolar) of analytes, even achieving single-molecule-level detection. SERS-based sensors have high sensitivity and resolution, but are susceptible to background signal interference during detection.

**Figure 9:**
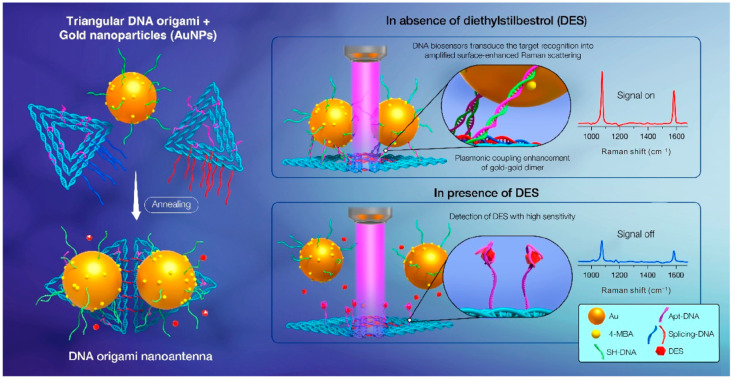
DNA origami and AuNP-assembled plasmonic dimer nanoantenna SERS biosensor used for DES detection. Reprinted with permission from ref. [115].

The presence of a localized electromagnetic field at the hotspot where two or more nanoparticles are connected can achieve SERS enhancements of up to 10^11^ [112]. The minimum structure required to obtain plasma hotspots is the nanoparticle dimer, and DNA origami can easily create a SERS device to accurately arrange nanoparticle dimers. At present, various DNA origami structures, such as rectangular and DNA-bridged rectangular origami and triangular and triangular dimer origami, have been used to assemble dimers such as AuNPs, Au nanobipyramids (Au NBPs) and Ag-coated Au nanostars to construct various SERS devices, achieving the label-free sensing and detection of a wide range of disease markers and biomolecules [111,112,113,114,115]. As an example, taking advantage of the DNA origami’s spatial addressability and the specificity of base pairing, Li et al. prepared a plasmonic dimer nanoantenna-based surface-enhanced Raman scattering (SERS) biosensor for the detection of trace amounts of diethylstilbestrol (DES) in food [115]. In this biosensor, the DNA origami was functionalized by aptamers: when the aptamer recognized and responded to the target, it induced a dynamic transition in the conformation of the plasmonic nanoantenna and output an amplified SERS signal (Figure 9).

The SERS effect is influenced by the number of nanoparticles in the assembly, the distribution of hotspots and the number of Raman molecules in the hotspots [116]. Fang et al. anchored a set of AuNPs with a diameter of 80 nm to a super-origami DNA framework assembled by triangular DNA origami and investigated the relationship between the number of fluorescent molecules accommodated in the hotspot region and the SERS intensity [117]. They found that the SERS intensity increased quantitatively with the number of ROX fluorescent molecules, and then it no longer significantly increases when it reached saturation. Niu et al. modulated the anchoring orientation of anisotropic AuNCs on DNA origami and assembled a variety of 2–4 multimer structures, including face-to-face (FTF), face-to-side (FTS) and side-to-side dimer (STS) orientations [118]. The plasmonic coupling and local electrical field enhancement characteristics of each structure hotspot region were investigated by finite difference time domain (FDTD) simulations and SERS. In addition, the dynamic assembly of nanoparticles led to enhanced SERS due to increased hotspots, which is also an effective strategy for the construction of highly sensitive SERS sensors. Xiong et al. developed a ratiometric SERS cytosensor for circulating tumor cell (CTC) detection [119]. They employed CTC to trigger the release of Zn^2+^-specific DNAzymes as DNA walkers, cleaving the H1 hairpin DNA structure in the presence of Zn^2+^. The cleaved fragments hybridized with the ssDNA modified on AuNPs to form network nanostructures (Nw NSs) and output strong SERS signals.

### 3.4. Other Sensors

Colorimetric sensors use color changes as the analysis basis and achieve the qualitative or quantitative detection of target objects by observing and analyzing the color changes caused by the aggregation or dispersion of Au NPs directly or indirectly triggered by the target objects [120]. With the advantages of simple operation, a low price and an intuitive nature, they have been widely used in the fields of environmental pollution, biochemical analysis and disease diagnosis, but the sensitivity is relatively low. For example, Kongpreecha et al. developed an aptamer sensor for the detection of paraquat pesticides [121]. Aptamers that could selectively interact with paraquat were covered on the AuNPs’ surfaces by non-covalent interaction in advance. In the absence of paraquat, AuNPs remained dispersed and showed a red color. When paraquat was present, the aptamer combined with paraquat led to the AuNP surface being unprotected, resulting in aggregation and generating a blue solution after the addition of NaCl.

Plasmonic sensors based on dark field imaging and scattering spectra have been used to analyze signals or color changes induced by targets at the individual particle scale with high sensitivity [122,123]. However, the scattering spectrum is directly related to the size, morphology and degree of aggregation of nanoparticles, which may lead to measurement biases among individual particles, and it is susceptible to background signal interference. For example, Zhou et al. used the AuNP dimer structure to establish a sensor for the ultra-sensitive detection of carcinoembryonic antigen (CEA) [124]. The individual AuNPs appeared as a green spot under a dark field microscope; in the presence of CEA, the distance between the antibody-specific labeled DNA-modified AuNPs diminished to form a dimer, and a yellow spot was observed under the dark field microscope. Highly sensitive immunodetection was achieved by colorimetry, counting and scattering spectroscopy analysis.

Lateral flow test strips (LFTSs) are a simple, low-cost, fast and portable but limited-sensitivity strip-sensing technique [125,126]. A liquid sample containing the analyte flows along the test strip driven by capillary action and interacts with a probe attached to the sensing area on the test strip to realize the detection and analysis of samples at millimolar or micromolar concentrations. For example, Zha’s group proposed a nanostructure-based stripe sensor. They used the DNA tetrahedral nanostructure as a scaffold for the capture probe and fixed the assembled capture probe in the sensing region. When a functional AuNP flowed through the sensing area, the capture probe was combined with the AuNPs. Due to the aggregation of AuNPs, a red line was observed. Through the design of targets and probes, they achieved the detection of exosomes microRNA-150-5p [127] and carbapenemase genes [128].

A temperature sensor is used to obtain and monitor temperature information, which requires a device with high heat sensitivity in the sensor. The hybridization and unwinding of DNA sequences are highly temperature-dependent. Altering the sequence length or G/C content, introducing mismatched bases, base stacking, etc., can lead to changes in the kinetics and efficiency of temperature-dependent DNA assemblies [129,130]. Therefore, DNA sequences can be designed as heat-sensitive devices in reconfigurable temperature sensors. For example, Johnson and co-workers developed a temperature-driven device based on a complex DNA origami system [64,131]. Complementary DNA sequences were designed and anchored inside the DNA origami hinge and on the AuNP surface. The overhang sequence was adenine with a length of 6–8 bases, which could respond to temperature changes. The hinge opened when the temperature increased and closed when the temperature decreased, and the device showed a significant response in the temperature range of 30–45 ℃. However, temperature sensors are susceptible to environmental factors.

A comparison of the characteristics (including basic principles, performance, merits and drawbacks) of the above sensors is presented in Table 1.

## 4. Conclusions

In summary, DNA, as a powerful programmable tool, has been widely used to regulate the high-order assembly of gold nanoparticles. This review first introduces the constructed component types and assembly strategies, including the use of DNA single strands, DNA tiles and DNA origami nanostructures to regulate the assembly of gold nanoparticles into multimers, 1D and 2D arrays and 3D lattice structures. Moreover, the highly controllable assembly of DNA-mediated gold nanoparticles has unique advantages in achieving external stimulus responses and signal amplification, and it has been widely applied in the sensing field. This review further discusses the related applications of DNA-assembled gold nanoparticle assemblies in the field of sensing from the perspectives of sensor construction strategies and detection technologies, including fluorescence-based sensing, chiral sensing, SERS-based sensing, etc.

The combination of DNA nanotechnology and gold nanoparticles has broadly improved the performance of gold nanomaterials. Firstly, the complexity of gold nanomaterials depends to a certain extent on the DNA nanostructure template. The development of DNA nanotechnology has made it easy to design diverse DNA nanostructure templates. Secondly, the spacing between gold nanoparticles affects their plasmonic coupling effect, and DNA strands with adjustable lengths and addressable DNA nanostructures have unique advantages in regulating the spacing between gold nanoparticles. Thirdly, the uncontrollable DNA modification density and position on the surfaces of gold nanoparticles affect the orientation of gold nanoparticle assemblies. Pattern transfer strategies provide a good solution for the accurate adjustment of the position and quantity of conjugated DNA. Fourthly, stimulus-responsive materials are an indispensable component in creating nanomachines such as dynamic chiral sensors. Benefitting from the unique properties of DNA, a wide range of sequence-specific materials that respond to external stimuli can be designed. Fifthly, in the biological application of gold nanoparticles, DNA can not only mediate the assembly of functional materials, but also endow gold nanomaterials with better biocompatibility.

Although DNA nanotechnology and gold nanoparticles can produce complex gold nanostructures with controllable functions and ideal self-assembly, there are still great challenges and limitations. Firstly, maintaining the stability of gold nanoparticles is a key issue. The presence of salt ions weakens the electrostatic repulsion between gold nanoparticles, leading to their instability and aggregation. This process occurs during the surface modification of DNA and the subsequent assembly of gold nanoparticles, leading to an increase in raw material costs and a decrease in yield. Secondly, high yields of target structures are usually required for assembler property exploration and application. Therefore, appropriate purification and post-processing are required, which will result in lower yields and the consumption of time and labor. Thirdly, in order to construct complex gold nanoparticle assembly structures (e.g., arrays), it is generally necessary to have a sufficiently large DNA structural template. The size of the folded origami structure obtained by a single scaffold is limited by the length of the scaffold, and high-order origami assembly with high difficulty and low yields is usually required. Fourthly, although various sensing technologies based on gold nanoparticle assemblies have been proposed, further exploration is needed to translate these technologies into practical sensing applications. Fifthly, the controllable and reversible construction of the assembly and disassembly process of gold nanoparticles is very important in the sensing field. However, as the number of reconstructions increases, the stability of the structure decreases. The question of how to maintain the high performance of functional devices without degradation is worth further research. Despite the challenges and limitations, we anticipate that DNA nanotechnology will continue to play an important role as a manufacturing platform for the assembly of complex plasmonic structures and will lead to exciting scientific and technological developments.

## Figures and Tables

**Table 1:** Comparison between different sensors.

Sensor	Principle	Performance	Merits	Drawbacks
Fluorescence-based sensor	Interact with target analytes, resulting in changes in fluorescence signals	Limit of detection (LOD): nanomolar to picomolar	High sensitivity, high selectivity, and real-time detection	Influenced by background interference and fluorescence quenching
Chiral sensor	Chiral structural changes lead to chiral changes	Chiral recognition LOD: nanomolar or lower	High sensitivity, high selectivity, high specificity, and real-time detection	Vulnerable to non-specific interference
SERS-based sensor	AuNP structure serves as SERS substrate to enhance detection signals	LOD: femtomolar to nanomolar or molecular level detection	High sensitivity, good resolution	Susceptible to background signal interference
Colorimetric sensor	AuNP aggregation or dispersion cause color changes	Visually visible color changes	Simple operation, no special equipment, and low cost	Relatively low sensitivity
Plasmonic sensor	Different particles have different signals for dark field imaging or scattering spectra	Single particle High-contrast imaging	Microscopic-scale observation with high sensitivity	Sample bias Susceptible to background signal interference
Lateral flow test strips (LFTSs)	Target object interacts with the probe attached on sensor, resulting in changes in color or signal	LOD: millimolar or micromolar	Simple and rapid detection, portable and low-cost	Limited sensitivity
Temperature sensor	Temperature dependence of DNA strands	Sensitive to temperature changes	Reusable	Susceptible to environmental factors

## Data Availability

Data are available in a publicly accessible repository.
